# Life Design Counseling: Theory, Methodology, Challenges, and Future Trends

**DOI:** 10.3389/fpsyg.2022.814458

**Published:** 2022-02-01

**Authors:** Ya Wen, Kai Li, Huaruo Chen, Fei Liu

**Affiliations:** ^1^School of Teacher Education, Nanjing Xiaozhuang University, Nanjing, China; ^2^School of Philosophy, Wuhan University, Wuhan, China; ^3^School of Education Science, Nanjing Normal University, Nanjing, China; ^4^Center for Research and Reform in Education, Johns Hopkins University, Baltimore, MD, United States; ^5^School of Education Science, Huaiyin Normal University, Huaian, China

**Keywords:** life design counseling, theory, methodology, challenges, trends

## Abstract

With the rapid development of society and the dramatic change of environment, previous career counseling focusing on personal choice has been difficult to meet individuals’ needs. It is very meaningful and valuable to introduce the ideology of Life Design Counseling (LDC). In this mini review, we introduce and analyze the theory and methodology of LDC. This review puts forward challenges in the field of LDC, including the lack of attention to clients from multiple backgrounds and professional counselors, the lack of diversified methods in the intervention process, and the lack of diversified research. The theoretical research, practical research, and the integration of theory and practice of LDC still need to be further concerned by researchers.

## Introduction

The current living environment changes faster than ever before, resulting in the trend of personal career development from static and linear to dynamic and nonlinear. Personal future work and employment are becoming more and more unstable in this world dominated by Volatility, Uncertainty, Complexity, and Ambiguity (VUCA) (Canzittu, 2020). Savickas et al. (2009) proposed the third paradigm of career intervention, which is called the “Life Design Paradigm,” to deal with the drastic changes in the current social environment (Savickas et al., 2009). Based on this paradigm, Life Design Counseling (LDC) refers to a kind of counseling that helps clients define their career as a story, narrate their personal working life with continuity and coherence, discover life themes, create life meaning, construct identity, formulate adaptive actions, and pursue the life expected by individuals (Savickas and Pouyaud, 2016; Venter and Maree, 2020; Wong, 2021). LDC aims to help clients explore possible careers, reshape personal narrative identity through meaning construction, clarify self-concept, establish a life purpose and create a meaningful life (Savickas, 2012; Wehmeyer et al., 2018; Wen et al., 2020a). Compared with other career counseling methods, LDC provides a new perspective for individual or group counseling to formulate long-term career planning and has the potential for cross-cultural research (Savickas et al., 2018). Therefore, this mini review aims to introduce the theory, methodology, challenges, and future trends of LDC, so that researchers engaged in this specific field can more effectively understand and master the research trends and future development trends in this field, and expand the application scope of LDC. In order to ensure the quality of the literature, LDC was used as the search term to conduct a full search on the Web of Science in this study. The search scope is limited to English articles. Specifically, the literature search was conducted from January 2000 to November 2021. In addition, this study only included standard research papers, excluding news, conferences and other types of documents.

## What Is Life Design Counseling?

Life Design Counseling is a kind of career intervention, which originates from Guichard’s self-construction theory and Savikcas’s career construction theory (Guichard, 2009; Maree, 2019). After entering the 21st century, individuals need to face more complex social relationships and work environments, especially to design their own lives. The premise of designing life is self-construction. In response to the above changes, Guichard put forward self-construction theory, which mainly points out that self-construction is a subjective identity system of past, present, and future (Guichard, 2009; Guichard et al., 2012). With the development of career theory, in order to explore career from a more integrated perspective, Savickas et al. proposed Career Construction Theory. Career Construction Theory attempts to look at individual career from an overall perspective, aiming to answer three core questions, namely “What kind of career will be established?” “How to construct a career?” and “Why build a career?” (Savickas, 2008; Savickas and Pouyaud, 2016). Specifically, “What kind of career will be established?” reflects the relationship between personal interests, abilities, values and career development from the perspective of social division of labor (Rudolph et al., 2019). “How to construct a career?” is manifested as the interaction between individuals and the environment in the current mobile society and flexible work organization, emphasizing that individuals reshape themselves in reflection and cope with external challenges based on their career adaptability. “Why build a career” is the fundamental issue of Career Construction Theory, which truly reflects that individuals regard their own careers as creating their own life stories and strung the stories into a line around the theme of life. In general, career is a macro narrative about the role of work in one’s life which was defined in career construction theory. Both those two theories involve social constructivism, emphasize the significance of understanding and intervening narrative thinking in the process of construction, and highlight the value of reflexivity based on individual in-depth thinking of past and present experience for individual behavior (Guichard, 2016b; Savickas, 2016). In short, whether it is the theory of Self-Construction Theory or Career Construction Theory, reflexivity emphasizes the formation of the individual based on current problems to guide the next step in life. However, there are also some differences between the two theories: self-construction theory focuses on the survival direction of individuals, emphasizes the dynamic development of individuals based on subjective identity formal system (SIFS), and constructs self-concept in the social environment (Guichard, 2016a). LDC based on Self-Construction Theory adopts a relatively open interview form, pays attention to the role of clients’ self-construction in meaning, identity, and future planning, focuses on helping clients reshape their narrative identity, and projects the new possibility of self-construction into their career role.Therefore, it is more suitable for teenagers or emerging adults (Di Fabio, 2014; Guichard, 2016a). While Career Construction Theory points out that career can be a dynamic construction process, and subjective self and external world can adapt to each other. LDC based on Career Construction Theory helps clients to focus on their own career through highly structured interviews and narration around personal interests and models, increase their control over their future career positioning, stimulate their curiosity to explore possible self and possible future scenes, and build their confidence in career development (Maree, 2014). Counselors help clients reconstruct the meaning, identity, and intention of life, as well as find the life theme and answer the meaning of life, which is more applicable for mature adults (Guichard, 2016a; Hartung and Santilli, 2018).

In summary, LDC means that counselors help clients give meaning to their lives and social relationships through various stories and find their understanding of life themes as well throughout this process (Cardoso et al., 2016b; Wong, 2021). LDC can be regarded as an intervention method combining Self-Construction and Career Construction, which reflects the integration of personal personality characteristics, development process and life story (Di Fabio and Maree, 2012; Wong, 2021). LDC emphasizes that individuals reflect on themselves and construct meaning based on continuous narration by considering the overall impact of the surrounding system, so as to generate expectations and motivation for the future (Cardoso et al., 2016a; Venter and Maree, 2020). Specifically, LDC focuses on people’s interaction between themselves and the environment, establishes life trajectory through narration, and considers educational investment, work and life roles and meaning in life, so as to deal with the uncertainty of the future (Nota et al., 2016; Cardoso et al., 2019). In short, life-long, holistic, contextual and preventive are important characteristics of LDC (Savickas et al., 2009; Venter and Maree, 2020). LDC aims to help clients reshape their lives through narration based on personal needs, interests, abilities and experiences (Maree, 2020b). Clients can rewrite their narrative identity, clarify their self-concept, establish life goals and explore possible life tracks (Savickas et al., 2009; Cardoso et al., 2019; Maree, 2020a).

## How to Use Life Design Counseling?

Life Design Counseling is an emerging method of career intervention. The research on LDC is mainly divided into three categories: process intervention, process evaluation, and results evaluation. According to these three categories, some pioneer scholars have made a comprehensive evaluation system of LDC.

### Process Intervention

The process intervention on LDC mainly includes Guichard’s Life and Career Design Dialogues (LCDD) and Career Construction Interview (CCI) (Pouyaud et al., 2016; Barclay et al., 2019; Wong, 2021). My Career Story (MCS) is a written expression tool developed based on CCI (Hartung and Santilli, 2018). In addition, Interpersonal process recall (IPR) is an important auxiliary method of LDC that is usually used to explore the experience of clients in the process of counseling dialogue (Cardoso et al., 2016a). Written exercises, career collages, and career portfolios are often combined with CCI creatively(Barclay, 2019).

Specifically, LCDD is a psychological counseling and intervention method which focuses on the dialogue between client and counselors to help the client understand the self-construction of subjective identity (Guichard, 2016b; Pouyaud et al., 2016). The dialogue took place in one-on-one interviews between clients and counselors lasting several weeks (Pouyaud et al., 2016). CCI designed by Savickas (2011) is widely used which is usually divided into three sessions (Cardoso et al., 2018). The first session is Career Construction Interview (Cardoso et al., 2018). After the client and counselor discussed their expectations for intervention, LDC focused on five aspects: role models; favorite magazines, TV programs, etc.; current favorite story; Motto to oneself; early recollections (Taylor et al., 2016; Taylor and Savickas, 2016). For example, clients are required to tell real or fictional role models. Counselors can discuss the similarities and differences of role models with clients, so as to reflect the clients’ self-concept, including how to treat their own identity and values. Interested magazines and TV programs reflect the relationship between clients’ interests and working environment. During the second session, counselors assist the client in constructing a story, which aims to provide continuity for the fragments of the client’s life in the previous stage. In the third session, the focus of the intervention is to assist the client to connect life themes with career planning (Cardoso et al., 2018). As an auxiliary means, IPR aims to explore the experience of clients in the interview process. Clients are required to watch the video replay of the interview. Counselors need to ask clients how to experience the important moment in the video, help clients analyze the emotional experience of the important moment, and promote the reflection of clients in the process of interpersonal interaction with counselors. In addition, in the process of CCI, clients can create career collages based on paper pasting. The counselor assists the client in creating “a collage” and takes the collage as a tool for meaning construction. The client describes what the client attaches importance to and desires in his life by pasting images representing personal models, pictures of his favorite magazines and TV programs, so as to help the client imagine and design the future and obtain the power to realize the future.

### Process Evaluation

The purpose of the process evaluation of LDC is to find out what changes have taken place in the counseling process. The process evaluation of LDC can be divided into the qualitative evaluation and quantitative evaluation. The focus of qualitative assessment is whether the clients have changed and what changes have taken place in the LDC process. To analyze the changes of clients, researchers also used quantitative methods to process evaluation.

Specifically, Innovative moments (IMs) are usually used as a sign of changes in clients (Cardoso et al., 2020). At present, for the qualitative evaluation of IMs in LDC, the representative is the innovative moments coding system (IMCS) developed by Cardoso et al. IMCS is a tool for tracking IMs. IMs in the counseling process reflects the non-linear change of the clients, which is often accompanied by the circular ambivalence reflecting the self motivation of the clients. Researchers often use IMCS together with qualitative tools to evaluate the changes in clients’ ambivalence during the counseling process. Representative tools include Return to the Problem Coding System (RPCS) and Ambivalence Coding System (ACS) (Da Silva et al., 2020). The former refers to that the client immediately returns after generating the innovation moment and re emphasizes the problematic self narration, which weakens the significance of IM. The latter is often used to identify clients who immediately turn to problematic self narration after describing the moment of innovation.

Many researchers have conducted comprehensive research on LDC combined both qualitative evaluation and quantitative evaluation. Giving full play to the advantages of qualitative evaluation and quantitative evaluation, some scholars also try to develop a comprehensive evaluation of LDC. For instance, Maree Career Matrix (MCM) is used to measure the clients’ interest and confidence in the successful pursuit of a variety of careers (Maree and Taylor, 2016). MCM with 19 categories showed good psychometric characteristics during the standardization process. In terms of the comprehensive evaluation, MCM shows good psychometric characteristics in the standardization process (Morgan and Ferreira, 2019). In addition, Maree (2007) developed the South African version of the qualitative questionnaire of Career Interest Profile (CIP) (Di Fabio and Maree, 2013), trying to construct narrative information from individual career stories.

### Results’ Evaluation

Results’ evaluation is to compare the status of clients after counseling with those before counseling to find out the differences. From the aspect of the quantitative evaluation, researchers found that the clients’ career uncertainty and career pending decreased, while their preparation for academic specialty and career decision-making, professional self-efficacy, career planning, professional identity, and professional control trajectory, life satisfaction increased (Barclay and Stoltz, 2016a,b; Nota et al., 2016; Cardoso et al., 2018; Di Maggio et al., 2021b). The above research shows that through the intervention of LDC, individuals can more accurately evaluate themselves, gather occupational information, select goals, make plans for the future, have more certainty about their career development, and improve their evaluation of their own quality of life. Still, there exist some discrepancies among different research. Some scholars found that career adaptability improved, and others assert that this variable did not change (Cardoso et al., 2018; Rudolph et al., 2019). These studies suggest that the impact of LDC on an individual’s career adaptability is a complex issue, and more studies are still needed to examine the effects of interventions in more detail. In general, how the LDC is operated can be shown in Table 1.

**TABLE 1:** How the LDC is operated.

Number	Category		Illustration
1	Process Intervention	Main methods	1. Career Construction Interview (CCI) 2. My Career Story (MCS) 3. Life and Career Design Dialogues (LCDD) 4. Interpersonal process recall (IPR)
		Additional ways	1. Written Exercises 2. Career Collages 3. Career Portfolios
2	Evaluation	Process Evaluation	1. Innovative Moments Coding System (IMCS) 2. Return to the Problem Coding System (RPCS) 3. Ambivalence coding system (ACS) 4. Career Interest Profile (CIP) 5. Maree Career Matrix (MCM)
		Results’ Evaluation	1. Vocational Certainty Scale (VCS) 2. The Career Maturity Inventory–Form C (CMI-FC) 3. Career Decision Self-Efficacy Scale–Short Form (CDSE-SF) 4. The Career Adapt-abilities Scale(CAAS)

## What Are the Challenges of Life Design Counseling?

### Lack of Attention to Clients From Multiple Backgrounds and Professional Counselors

On the one hand, LDC needs to pay attention to the psychological needs of more diverse cultural background groups, because the counseling needs of different types of clients are unique and different (Sampaio et al., 2021). Scholars have accumulated some group LDC research in the early stage, such as the research on college students (Pordelan et al., 2018), middle and high school students (Nota et al., 2016), Italian entrepreneurs (Di Fabio and Maree, 2012), etc. However, these groups are still limited, and the research group based on LDC needs to be further expanded. For example, LDC may become an effective way to help vulnerable groups develop their career. On the other hand, the effective implementation of LDC is inseparable from professional counselors. The lack of professional LDC counselors may bring the following negative effects: it is difficult for clients and counselors to establish a working alliance and sort out ambivalence through coherent narration and re conceptualization, then it is hard for clients to draw a life portrait and formulate a plan for moving forward (Cardoso et al., 2016a,b; Barclay, 2019). In addition, long-term intervention is an important guarantee for the effectiveness of LDC (Di Fabio and Maree, 2012; Nota et al., 2016). If there is no professional LDC counselors, the continuous participation of clients may be reduced (Barclay and Stoltz, 2016a). Therefore, in the practice of LDC, it is particularly necessary to cultivate a large number of professional counselors who are familiar with the operation mode of LDC.

### Lack of Diversified Methods in the Intervention Process

The intervention methods for LDC are still not diversified enough. The typical intervention methods for LDC only include CCI, MCS, LCDD, etc. The combination of LDC and other practical methods can give better play to the function of career counseling. For example, in view of the close relationship between career and psychosocial issues, how to combine LDC and psychotherapy to solve more complex life problems of clients is a topic worthy of attention (Cardoso, 2016). In addition, the creative use of LDC is still a problem that needs the attention of career practitioners. Specifically, how to combine intervention methods of methods with art (e.g., painting) to help clients better narrate, reconstruct their own life themes, and find the power for change is a issue that needs to be solved urgently (Barclay, 2019). LDC practitioners need to creatively develop intervention methods, so that they can be applied to a wider range of clients, help them express themselves through narration, generate motivation for substantial change, discover life themes, find life goals and plan their own future. In general, counselors need to creatively and flexibly use CCI and other major intervention methods to help clients build their future careers.

### Lack of Diversified Evaluation Methods

Scholars lack research on the diversification of LDC in the existing research, especially in its evaluation method. First, the evaluation object is not comprehensive enough. Current research mainly focuses on the counseling effect on clients in LDC, while neglecting the different influence from counselors who plays the same important roles as well. Although clients are an important part of the counseling subject, the evaluation of counselors has a specific value, and researchers lack attention to the growth of counselors’ counseling ability (Storlie et al., 2017). The second is the evaluation method: the current research mainly includes qualitative evaluation, quantitative evaluation, and comprehensive evaluation (McMahon et al., 2003; Di Fabio and Maree, 2013). However, these evaluation methods are limited and the evaluation content is not rich. It seems that the evaluation of LDC can be further improved by referring to other counseling and evaluation methods as well as other evaluation contents.

## Future Research Trends

Although LDC is a relatively new way of career counseling and opens up new ideas for counselors, the theoretical research, practical research, and the integration of theory and practice of LDC still need to be further concerned and promoted by researchers. The main trends of future development of LDC are shown in Table 2.

**TABLE 2:** Future research trends of LDC.

Number	Item
1. Strengthen the in-depth study of clients and counselors	1. More vulnerable groups 2. The general population with finer classification 3. Improve the quality of counselors 4. Increase the number of counselors 5. Create a standardized process for counselor training 6. Establishment a working alliance between client and counselor
2. Extended intervention Methods	1. Pay attention to the cutting-edge trends in the field of counseling methods 2. Combine the methods in LDC with art therapy methods 3. Online and offline intervention
3. Improve intervention evaluation methods	1. Learn from other disciplines and develop more process assessment tools, such as big data, NVivo, etc. 2. From the perspective of personality characteristics, such as career optimism, mindfulness, gratitude, benevolence, etc. 3. From the perspective of organizational contexts, such as decent work, job satisfaction, job burnout, thriving at work, reemployment, etc 4. Clarify the relationship between counselors and researchers in the evaluation process and focus on the evaluation of counselors

### Strengthen the Needs Analysis of Clients and the Professional Training of Counselors of Life Design Counseling

Scholars need to create diversified counseling methods to broaden the application scope of LDC. On one hand, scholars especially need to pay more attention to the following groups: (a) vulnerable groups, such as the unemployed, people with disabilities, people who have experienced breakups or divorces, cancer patients, aging Chinese parents who lose their only child, patients with depression, survivors of family violence, refugees, ex-offenders, solitary person, the bereaved and the low-income people in remote areas (Nota et al., 2014; Li, 2018; Bergeron et al., 2021; Di Maggio et al., 2021a; Maree, 2021) and (b) the general population with finer classification, such as public or private primary school students in different countries or regions, middle school students from different regions and nationalities, high school students with different school characteristics, college students of different majors, workers of different occupations (Cardoso et al., 2016a; Maree, 2020a). Researchers may help these people achieve better career development by designing diversified LDC studies. On the other hand, more professional LDC counselors need to be trained, including improving the quality of counselors, increasing the number of counselors, and forming a complete and standardized process for training counselors, to serve more people who need LDC help (Di Fabio and Maree, 2012; Venter and Maree, 2020; Wen et al., 2020a). Researchers and practitioners need to pay particular attention to the formation and maintenance of a good relationship between counselors and clients, because the establishment of a working alliance between them is an important guarantee for effective LDC (Cardoso et al., 2016a,^2021^; Tian et al., 2020).

### Increase Multi-Dimensional and Diversified Intervention Process Methods of Life Design Counseling

In order to solve the lack of diversified LDC intervention methods, career counseling practitioners can try the following paths. First, LDC counselors can integrate other counselor theories with LDC and find the coincidence points between some cutting-edge psychology and pedagogy theories and LDC, so as to create a diversified counseling model and serve more clients with different needs. For example, future researchers can try to combine PERMA (Positive Emotions, Engagement, Relationships, Meaning, Achievements) theory in positive psychology with LDC, so as to jointly promote the positive change of clients (Carreno et al., 2021). Second, the existing LDC methods can be presented in a diversified and creative way in order to promote the self-expression and reflexivity of clients. Future career researchers and practitioners can try to combine the methods in LDC with art therapy methods such as painting, music, dance, and writing, so as to serve a more diverse group of individual or group clients. Last but not least, scholars may be able to conduct online and offline LDC through computers, mobile phones, telephones, and e-mails (Savarese et al., 2020; Carbone et al., 2021), and other flexible forms and combined with other emerging psychological counseling methods. In general, career practitioners need to create diversified LDC intervention methods in the future to help clients through narrative construction, deconstruction and joint construction of their personal career stories, find the core life theme, and carry out a meaningful life.

### Strengthen the Research on the Evaluation Objects and Methods of Life Design Counseling

Future research on LDC needs to enhance the research on the evaluation objects and methods of LDC. Strengthening the research on the evaluation is mainly reflected in the following three points: Firstly, researchers need to further broaden the application scope of the above evaluation methods and also create more process evaluation methods suitable for LDC because of big data, Nvivo to serve a wider group (Alam, 2020; Wen et al., 2020a). Secondly, in terms of personality characteristics, it should be paid more attention to such things as the meaning of life, mindfulness, and optimism (Ginevra et al., 2018; Wen et al., 2020b). From the perspective of the organizational situation, it should focus on exploring the influence mechanisms caused by self and the outside world, such as professional identity, job satisfaction, social support, and job burnout (Chen et al., 2020; Shen et al., 2021). Finally, strengthen the research on counselors in the evaluation of LDC. Scholars need to further enrich the evaluation objects of LDC and pay attention to the evaluation of counselors. Previous studies usually focused on the evaluation of clients. However, it needs to pay more attention to the uniqueness of counselors and the important value of counselors’ personal growth to the counseling effect, such as counselors’ cultural background, personality, value, and helping counselor trainees’ personal growth and professional development through supervision (Prosek and Michel, 2016). At the same time, since researchers and counselors may be the same group in some studies, future researchers need to further clarify the responsibilities and authorities of counselors and researchers in the LDC evaluation process (Tian et al., 2020).

## Conclusion

Life Design Counseling is a new form of career counseling in the VUCA era. The emergence and development of LDC are based on Self-Construction Theory and Career Construction Theory. At present, the use of LDC mainly includes process intervention, process evaluation, and results evaluation. To sum up, this study found some challenges of LDC, including lack of attention to clients from multiple backgrounds and professional counselors, lack of diversified methods in the intervention process, and lack of diversified evaluation methods. Therefore, this study came up with some suggestions about how to deal with those challenges as follows: Firstly, facing the changeable environment, future research should focus on strengthen the needs analysis of clients and the professional training of counselors of LDC. Secondly, for more individuals or groups to obtain career development through LDC, it is necessary to increase the research on the multi-dimensional and diversified intervention methods of LDC in future research. Finally, future researchers need to pay attention to strengthening the research on the evaluation objects and methods of LDC to ensure further prove the effect of LDC on individuals or groups.

## Author Contributions

All authors participated in the study design. YW and HC wrote the first draft. KL and FL modified the manuscript.

## Conflict of Interest

The authors declare that the research was conducted in the absence of any commercial or financial relationships that could be construed as a potential conflict of interest.

## Publisher’s Note

All claims expressed in this article are solely those of the authors and do not necessarily represent those of their affiliated organizations, or those of the publisher, the editors and the reviewers. Any product that may be evaluated in this article, or claim that may be made by its manufacturer, is not guaranteed or endorsed by the publisher.
